# Catheter ablation of typical atrial flutter with the superior approach in a patient with inferior vena cava interruption

**DOI:** 10.1002/ccr3.1196

**Published:** 2017-09-26

**Authors:** Vassil Traykov, Daniel Marchov, Galina Kirova, Valeri Gelev

**Affiliations:** ^1^ Department of Invasive Electrophysiology and Pacing Clinic of Cardiology Acibadem City Clinic Tokuda Hospital Sofia Bulgaria; ^2^ Department of Radiology Acibadem City Clinic Tokuda Hospital Sofia Bulgaria; ^3^ Department of Invasive Cardiology Clinic of Cardiology Acibadem City Clinic Tokuda Hospital Sofia Bulgaria

**Keywords:** Atrial flutter, catheter ablation, congenital heart disease, inferior vena cava interruption, subclavian vein approach

## Abstract

Inferior vena cava (IVC) interruption is a rare condition that might pose difficulties during typical flutter ablation. When azygos vein continuation is present ablation via the femoral route could be performed. In the absence of azygos vein continuation, typical atrial flutter ablation via a superior approach from the SVC is feasible.

## Introduction

Inferior vena cava (IVC) interruption is a rare anomaly that might be associated with other congenital abnormalities 1. The suprarenal segment of the IVC is most commonly affected, and the venous blood from the lower parts of the body is drained to the right atrium (RA) via the azygos vein in most cases. Catheter ablation of atrial arrhythmias requires femoral venous access. In the presence of IVC interruption, the procedure might be technically challenging and require alternative approach to the right heart.

## Case History, Investigations, and Treatment

**Figure 1:**
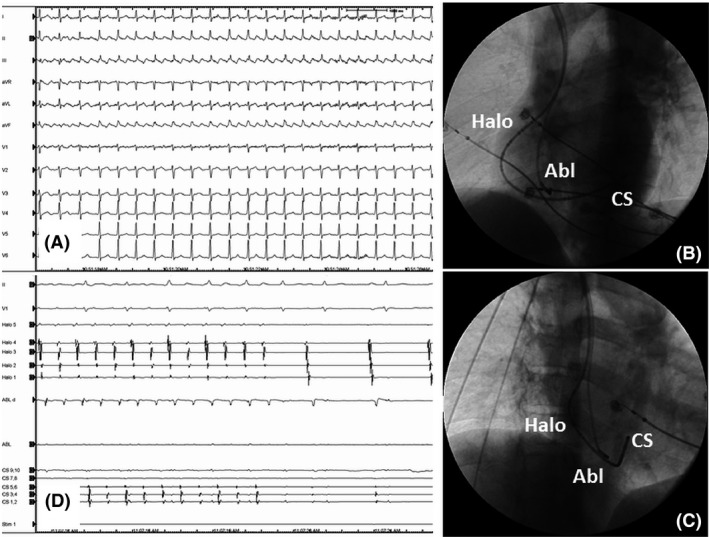
(Panel A) Twelve lead preprocedural ECG demonstrating typical counterclockwise atrial flutter with negative sawtooth F waves in the inferior leads. Panels B and C. Fluoroscopic catheter position in 45° left anterior oblique projection (Panel B) and in 30° right anterior oblique projection (Panel C). A duodecapolar catheter with its proximal five bipoles into the CS. The five proximal bipoles are positioned at the lateral tricuspid annulus. Ablation catheter is at the cavotricuspid isthmus. (Panel D) Surface ECG leads I, II, and V1 and intracardiac electrograms from the coronary sinus, lateral tricuspid annulus, and the ablation catheter at the cavotricuspid isthmus demonstrating atrial flutter termination during radiofrequency application at the cavotricuspid isthmus. Abl, ablation catheter positioned at the cavotricuspid isthmus; CS, coronary sinus; Halo, bipoles positioned at the lateral tricuspid annulus. Sweep speed: 25 mm/sec for panel A and 50 mm/sec for panel C.

A 52‐year‐old man was diagnosed with typical atrial flutter and referred for ablation at our center. He was symptomatic with palpitations and shortness of breath on exertion. There was no history of heart disease, and physical examination was unremarkable except for the presence of tiny mesh of dilated small cutaneous veins in the pelvic region. His presenting ECG showed typical sawtooth F waves in the inferior leads (Fig. 1, panel A). Preprocedural workup found no evidence of structural heart disease, and the patient was scheduled for catheter ablation.

Under local anesthesia right sided femoral venous access was easily acquired using the Seldinger technique. The planned procedural catheter setting was two 6F steerable diagnostic decapolar catheters—one of them to be positioned in the CS and the other at the lateral tricuspid annulus (TA) with the distal bipole positioned close to the lateral cavotricuspid isthmus (CTI) and a 7F ablation catheter with an 8‐mm distal tip. None of these catheters could be advanced to the IVC with the right sided venous access. Catheter advancement was held at the level of the right iliac vein. Then angiography was carried out with 20 mL of iodine contrast that demonstrated venous interruption of the right iliac vein at the confluence with the IVC. Marked collateralization was also noted. Subsequently, left femoral venous access was carried out and the angiography carried out through this access also demonstrated venous interruption of the left iliac vein at the confluence with IVC. Iodine contrast was seen to opacify the hepatic segment of the IVC via massive abdominal collaterals. Due to inability to access the RA via the femoral route, a double right sided subclavian venous access was acquired uneventfully with two 7F sheaths. A 7F duodecapolar diagnostic catheter (Duo‐decapolar, Biosense Webster, Diamond Bar, CA) and a 7F ablation catheter with 8‐mm distal tip (SJM Therapy, St. Jude Medical, St. Paul, MN) were introduced into the RA. The duodecapolar catheter was positioned with its distal five bipoles into the coronary sinus (CS). The most proximal of these bipoles was positioned at the CS ostium as assessed in the best left anterior oblique (LAO) projection. The proximal five bipoles were positioned at the lateral TA (Fig. 1, panels B and C).

With the above described catheter setting atrial flutter with a CL of 255 msec was diagnosed. The CS was activated from proximal to distal, and the lateral TA was activated from cranial to caudal. The CTI was found to be a part of the reentrant circuit by entrainment. So were CS ostium, posterolateral, and lateral TA. Therefore, typical atrial flutter was diagnosed. CTI ablation guided by the maximum voltage technique 2 was carried out by delivering 16 lesions with a target temperature of 70°C and target power of 70 W. Atrial flutter terminated during ablation (Fig. 1, panel D) and bidirectional CTI block was established. Total procedure duration was 96 min with fluoroscopy time of 23 min.

**Figure 2:**
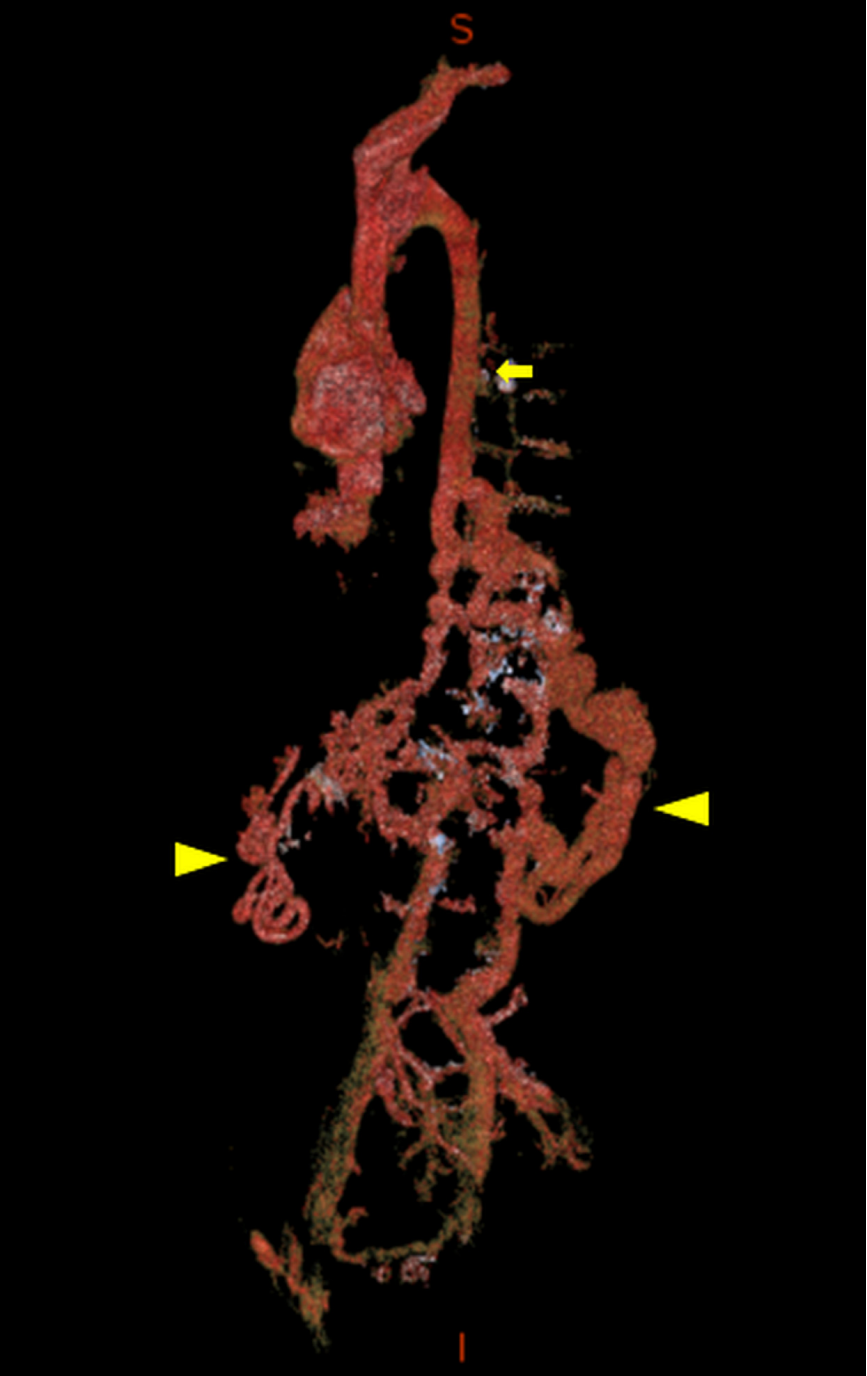
Three‐dimensional volume rendering of the venous angiography presented in shallow left anterior oblique projection. IVC is absent in its inferior segments. There are well‐developed intra‐abdominal collaterals (arrowheads). The blood from the inferior parts of the body is drained via the paravertebral veins and the intra‐abdominal collaterals to the dilated azygos vein (arrow).

Contrast‐enhanced MDCT was performed following the ablation in this patient. It demonstrated absence of the subrenal, renal, and suprarenal IVC segments. The venous blood from the inferior parts of the body was shown to be drained to the dilated azygos vein via hemiazygos vein and the paravertebral veins (Fig. 2). In addition, there were collaterals between the renal veins and the portal systems. The subatrial segment of IVC with the hepatic veins was normally developed.

At 9 months' follow‐up, the patient is asymptomatic with no arrhythmia documented on serial Holter recordings.

## Discussion

Inferior vena cava interruption is a rare condition that occurs in 0.6% of patients with congenital heart disease and even less frequently in the overall population 1. It may be associated with numerous other abnormalities. The suprarenal segment is most commonly affected, and venous blood drainage from the lower parts of the body is carried out via the azygos/hemiazygos vein continuation. In some cases, the latter is not well developed, and venous return to the RA is carried out via intra‐abdominal collaterals.

Electrophysiological procedures usually require femoral venous access. Abnormalities of the IVC pose technical difficulties to the procedure. In the presence of azygos vein continuation, catheter ablation via the femoral route could still be performed successfully, although at the expense of impaired catheter maneuverability and stability 3, 4. In its absence, alternative routes to access the right heart should be sought. These include internal jugular, subclavian, or even transhepatic access 4. In our case, several segments of the IVC were not developed, and the access to the azygos vein was impossible. Therefore, the right subclavian vein was used successfully for the ablation of counterclockwise typical atrial flutter. This route provides a reliable access to the right heart certainly at the expense of catheter stability. Navigating the catheter to the CTI and providing a stable and firm catheter contact at that region were quite challenging in the reported case. This was felt to be due to the need to position the catheter with an almost straight shaft that prevented the operator from being able to navigate the catheter precisely to the whole CTI region. To the best of our knowledge, there is only one case reported so far of successful CTI ablation using an approach from the superior vena cava 5.

## Conclusions

Catheter ablation of the CTI using a superior approach in patients with IVC interruption is feasible. Catheter navigation to the CTI and contact at the ablation sites might be challenging with this approach.

## Authorship

VT: concept and manuscript preparation. DM: involved in manuscript preparation. GK: involved in manuscript preparation and critical review of the manuscript. VG: concept and critical review of the manuscript.

## Conflict of Interest

None, relevant to the current work.
